# Use of Technology to Identify and Cover Underweighted Topics in Resident Conference Curriculum

**DOI:** 10.7759/cureus.3967

**Published:** 2019-01-26

**Authors:** Asit Misra, Charlotte Lawson

**Affiliations:** 1 Emergency Medicine, OhioHealth, Columbus, USA; 2 Emergency Medicine, University of South Carolina School of Medicine, Greenville, USA

**Keywords:** emergency medicine, graduate medical education, resident education, residency, curriculum improvement, curriculum evaluation, educational resource, international emergency medicine, free open access medical education (foam)

## Abstract

Emergency medicine training programs face many challenges in creating and maintaining high quality didactic and asynchronous learning experiences. To address these challenges, our team created two tools. First, we designed the Emergency Medicine Curriculum Assessment Tool (EMCAT) to help program leaders compare their didactic program to the Model of Clinical Practice established by the American Board of Emergency Medicine (ABEM). Second, we created a catalog of free, open-access medical education (FOAMed) resources based on the ABEM Model subcategory. Residency leaders can use EMCAT to identify the underweighted topics in their conference program and then access the resource catalog to find educational content matched to their areas of increased need. To date, five programs have implemented EMCAT and users from over 72 countries have accessed nearly 1,000 resources. Both EMCAT and the resource catalog are available free online.

## Introduction

Emergency medicine (EM) is a relatively new specialty with additional training programs continuing to form both in the United States and abroad [1-3]. Didactic and asynchronous education represents a pivotal component to formalized residency programs [4-7]. In the United States, the Accreditation Council for Graduate Medical Education (ACGME) mandates that five hours per week of resident education occur in a didactic and/or asynchronous setting [8,9]. New as well as established EM residency programs face challenges in developing and maintaining high-quality educational experiences over time. Some of the examples are faculty engagement, the difficulty of implementing more creative or innovative teaching methods, and the ability to find areas of redundancy or gaps in content coverage systematically. We sought to develop two tools that could help address these issues and offered them to use for free at www.mededguru.org.

## Technical report

Objectives

1) Our primary objective was to help emergency medicine residency programs in identifying under/overweighted areas of their conference curricula by offering the Emergency Medicine Curriculum Assessment Tool.

2) Our secondary objective was to decrease barriers to faculty engagement in didactic education by providing a starting point for otherwise labor-intensive in-person activities by an online resource catalog.

Curricular design

Audience

Emergency medicine residency program leaders and educators seeking a starting point for didactic curriculum development.

Rationale

Emergency Medicine Curriculum Assessment Tool (EMCAT) uses an online relational database application AirTable™. This medium looks like the spreadsheets that are familiar to most academicians. AirTable™ allows several fields having relationships to many items (as opposed to the “one-to-many” structure of programs like Microsoft Excel™). This is advantageous due to increased data integrity and the ability to quickly examine multiple facets of a dataset at once. Our tool helps to import years worth of curriculum data retrospectively from programs like Microsoft Excel™. It also allows setting up seamless, automated prospective data collection by integrating it with existing methods to keep track of conference schedules such as Excel™, Sheets™, and Google Calendar™, etc.

Description

We created the EMCAT to help emergency medicine residency programs assess their didactic curricula in comparison with the American Board of Emergency Medicine (ABEM) Model of Clinical Practice [10]. To supplement EMCAT, we also curated an online resource catalog of high-quality emergency medicine resources selected on the criteria for social media-based scholarship in health professions education [11].

EMCAT can be used retrospectively or prospectively to assess existing or plan future curricula. Prospective use is simple: program leaders enter their future educational sessions directly into the tool and code it with the appropriate date/time, format (lecture, small group, simulation, etc.), and topic (from the list of ABEM Model subcategories). An option of recording additional information such as attachments, speaker name, and session location is also available.

Using EMCAT retrospectively requires leaders to assemble their existing curriculum, usually in the form of their conference schedule, with supplementary material such as Simulation and Journal Club topics, etc. Once information is uploaded to EMCAT, each session needs the coding with one or more ABEM Model of Clinical Practice subcategories.

In either case, to allow separate views EMCAT is pre-programmed to group the provided data in different ways. Selecting the “Grouped by ABEM Model Subcategory” view allows leaders to identify both areas of redundancy and gaps in coverage quickly. With their identified gaps in hand, program leaders can then use the resource catalog at www.mededguru.org to “plug the holes” in their existing educational program by browsing for the specific ABEM Model subcategories in which their curriculum lacks coverage.

## Discussion

We designed EMCAT for the EM residency program leaders around the world who seek to improve their off-shift curriculum (i.e., didactics, simulation, journal club). EMCAT allows program leaders to easily compare their existing curricula to the ABEM Model of Clinical Practice, to identify gaps or underweighted areas, and to fill the gaps by accessing high-quality emergency medicine educational resources online. It is also essential for faculty members to have a variety of educational resources that they can choose from and use to teach. Our resource catalog provides a collection of simulation cases, resources for the flipped classroom and variety of Free Open Access Meducation (FOAMed) resources screened for quality and content which is easily accessible to all, including the audience from low- and mid-income countries where EM is still developing as a specialty and the faculty members do not have access to high-quality educational resources that they can use to teach the residents. Our resource catalog provides different types of filters to help the users find the exact resource type. We also wanted to provide a starting platform for the learners and educators to use FOAMed effectively.

**Figure 1:**
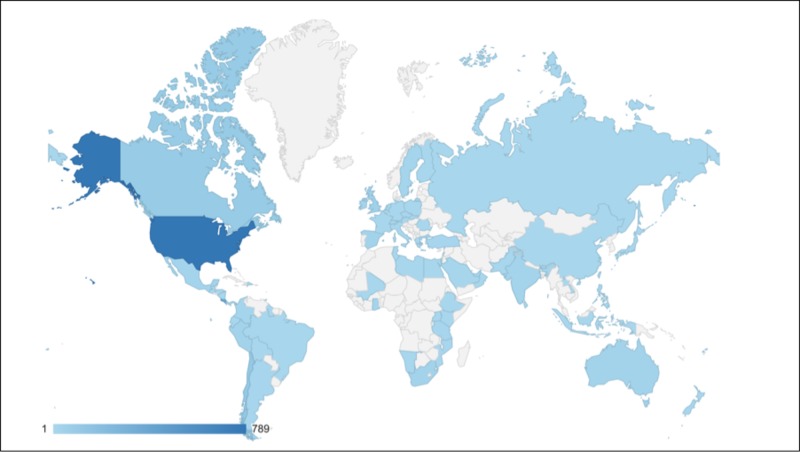
Map of users.

Since the soft launch of mededguru.org (March 2018 through July 2018), we have had 9,870 page views from users in 72 countries (Figure 1). Five residency programs are actively using EMCAT to track their residency program’s progress, and all are in the United States.

## Conclusions

We feel that our EMCAT tool will help residency programs to find any gaps in their current curriculum in the United States and with little modification we will be able to offer this tool to emergency medicine residencies in other countries. On the other hand, our resource catalog is continuously screened and updated for high-quality educational resources and is available to help educators, emergency medicine faculty members, residents, medical students, physician assistants and nurses nationally and internationally.
